# Asthma costs and social impact

**DOI:** 10.1186/s40733-016-0029-3

**Published:** 2017-01-06

**Authors:** Carlos Nunes, Ana Margarida Pereira, Mário Morais-Almeida

**Affiliations:** 1 Algarve Immunoallergy Center, Portimão, Portugal; 2 Allergy Center, CUF Descobertas Hospital, Lisbon, Portugal

**Keywords:** Asthma, Burden, Control, Costs, Morbidity, Mortality, Socio-economic

## Abstract

In recent decades, both asthma prevalence and incidence have been increasing worldwide, not only due to the genetic background, but mainly because of the effect of a wide number of environmental and lifestyle risk factors.

In many countries noncommunicable diseases, like asthma, are not yet considered a healthcare priority. This review will analyze and discuss disparities in asthma management in several countries and regions, such as access to healthcare human resources and medications, due to limited financial capacity to develop strategies to control and prevent this chronic disease.

This review tries to explore the social and economic burden of asthma impact on society. Although asthma is generally accepted as a costly illness, the total costs to society (direct, indirect and intangible asthma costs) are difficult to estimate, mainly due to different disease definitions and characterizations but also to the use of different methodologies to assess the asthma socio-economic impact in different societies.

The asthma costs are very variables from country to country, however we can estimate that a mean cost per patient per year, including all asthmatics (intermittent, mild, moderate and severe asthma) in Europe is $USD 1,900, which seems lower than USA, estimated mean $USD 3,100.

## Background

In recent decades, both asthma prevalence and incidence have been increasing worldwide, not only due to the genetic background, but mainly to the effect of a wide number of environmental risk factors, many of which included under the umbrella of “modern lifestyle”. However, the worldwide economic globalization could also have contributed to a significant increase in the asthma burden, even in developing countries that presented a low disease prevalence in the recent past [1, 2].

In this review, we will analyze and discuss some items regarding the socio-economic aspects of asthma, such as:

- The burden of asthma
- Asthma as a global disease
- Asthma mortality
- Asthma characterization
- Asthma co-morbidities
- Global asthma cost (direct and indirect)
- Social impact

### The burden of asthma

Asthma is primarily a chronic inflammatory disease that tends to present as a lifelong condition, with different severity degrees throughout the asthma patient’s life [1, 2]. Described since Hippocrates, asthma affects people from all age groups and presents its peak incidence in childhood. Recent data from the general population showed that in children up to 5 years old, the overall asthma incidence rate was 23/1,000 children per year; this incidence rate decreased among youth aged 12–17 years old to 4,4/1,000/year. Adult females had 1.8 times greater asthma incidence than adult males (4.9/1000 vs. 2.8/1000, respectively) [3].

In the United States of America (USA), per the National Health Interview Survey (NHIS)-2012, about 40 million people suffered from lifetime asthma (13% of the USA population) and 26 million people (8%) suffered from current asthma [4]. The lifetime asthma prevalence in different countries was estimated to range from 1 to 18% of the general population [5].

So, worldwide, it is estimated that nowadays more than 300 million people have asthma [1]. In 2025, we will probably have more 100 million asthma patients [5]. However, this may be an underestimation as asthma underdiagnosis is well-known [1]. Moreover, estimates of asthma prevalence are highly variable per the different methodologies used to collect and report data (e.g. different asthma definitions, different outcomes, among others). In fact, the best combination of questions to define asthma in epidemiological studies is not known. A recent review summarized the operational asthma definitions used in prevalence studies and evaluated how asthma prevalence estimates vary depending on the definition used [6]. In the literature, lifetime asthma, diagnosed asthma and current asthma were defined in 8, 12 and 29 different ways, respectively. By applying definitions of current asthma to data from the 2010 Portuguese National Asthma Survey (INAsma) [7] and the 2005-2006 National Health and Nutrition Examination Survey (NHANES) [8], the prevalence ranged between 5.3 to 24.4% and 1.1 to 17.2%, respectively. The authors concluded that epidemiological asthma definitions can lead to highly variable estimates of asthma prevalence and a standardized operational definition is needed [6].

Asthma is associated with a high disease burden. In children aged less than 5 years old and as well as in the mid-childhood ages, 5–14 years old there is a high prevalence worldwide with a significant relevance and even in these age groups is consider as one of the top chronic conditions causing disability-adjusted life years (DALYs). There are striking global variations in the prevalence of asthma symptoms in children, with an up to 13-fold difference between countries [1, 5]. Although asthma has a high burden in children, the relative importance of asthma impact increases with age and is particularly apparent in elderly, especially in women [9]. In Korea, for instance, although the costs for children accounted for the largest proportion of the total, the per capita cost was highest among patients with ≥50 years old [10].

Asthma symptoms are usually more common in high-income countries (HICs); however, some low- and middle-income countries (LMICs) also have a high prevalence [1, 5]. In fact, the highest prevalence of severe asthma symptoms among children with wheeze in the past 12 months is found in LMICs [11]. From the 1990s to the 2000s, asthma symptoms became more common in some high-prevalence centers in HICs; however, in many HICs, the prevalence remained stable or even decreased [12]. Concurrently, many LMICs with large populations showed prevalence increases, suggesting that the overall world asthma burden is increasing, while global disparities in asthma prevalence are decreasing ([11, 12].

Asthma can be deemed a significant public health problem, which often requires the use of emergency care, sometimes including hospital admission, and is responsible for a high number of missed school and/or work days; moreover, it can cause early permanent disability and premature death [5, 11]. In fact, asthma can be associated with significant limitations on physical, social and professional/student aspects of the life of those who suffer from this disease, namely when it is not controlled [1, 5]. Overall, asthma-related costs are very high [12].

Recent data from USA clearly showed that medical expenditures attributable to asthma were significantly higher for those with markers of uncontrolled disease when compared with medical expenditures of those who did not have asthma. Moreover, individuals with uncontrolled asthma, when compared with those without asthma, had up to 4.6-fold greater frequency of hospitalizations (*p* < 0.01), up to 1.8-fold higher number of emergency department visits (*p* < 0.01) and lower productivity (more likely to be unemployed, more days absent from work and more activity limitations; p < 0.01); however, hospitalizations rate of individuals with controlled asthma were not different from heathy subjects [13]. Similar results were found in several other surveys, namely in European asthmatic adults [14]. As the cost of asthma drastically increases as disease control decreases, substantial cost savings could be obtained through the proper management of asthmatic patients [1, 13].

### Asthma as a global disease

Asthma has clearly shown to be a global disease, however in last two decades was defined as a real public health problem affecting countries from all over the world and population of all age groups [1, 5, 15]. However, there are differences among countries, with rates significantly above the average, for example, in some native English-speaking countries (e.g.: UK, Australia and New Zealand) and, in contrast, much lower-than-average prevalence rates in some African and Asian countries [1, 15, 16].

Upon the first epidemiological publications on asthma, it was noted that asthma prevalence was higher in social classes with a high or very high annual income and that asthma severity was higher among the most disadvantaged. However, the latest epidemiological data from Africa, Latin America and Asia, showed that, in areas with low economic development, asthma prevalence has been increasing [1, 5, 16]. Although there could be several explanations, the development of larger cities, with consequent reduction of rural areas, may have played a role. With most the world population living in urban areas, the environmental conditions as the lifestyle changes have certainly influenced the asthma prevalence rate increase [2, 5].

Consequently, it can be said that asthma, worldwide, is “globalized and affect all countries as a public health problem”.

### Asthma mortality

The worldwide number of asthma deaths is about 180,000 per year, with a wide variation between continents, regions, age and economic groups [2, 16].

In the last decades, there was a significant reduction in asthma-related mortality, while, with an aging population, chronic respiratory diseases are becoming a more prominent cause of disability [17]. In fact, with the spread of new treatment guidelines, that emphasize the use of preventive anti-inflammatory drugs (e.g.: inhaled corticosteroids) to control the disease, mortality due to asthma has fallen substantially in most high-income areas; the USA is an exception, however, as no significant reduction on asthma mortality was seen in the last decades, especially in asthma patients with low-income [1, 2, 5, 17, 18]. Both premature deaths and permanent disabilities are costly, especially for those countries where these situations are more common [2, 5].

### Asthma characterization

Asthma is a complex chronic disease that presents acute exacerbation periods with dyspnea and bronchospasm which are usually reversible by bronchodilators [1]. Considering disease manifestations, patients with asthma can have both short-term treatment for asthma exacerbations and long-term treatment to obtain and maintain asthma control. Long-term treatment is crucial to reduce future risks, namely asthma attacks and lung function deterioration. Also, atopic sensitization and comorbidities are present in a large percentage of asthmatics. Indoor and outdoor allergies are very common and many asthmatics have more than one type of allergic sensitization.

Emergency care for the treatment of asthma attacks is frequently, and sometimes almost exclusively, used by many asthma patients [2, 5]. This frequent need for asthma-related unscheduled medical observations has multiple identified causes, namely reduced compliance or non-compliance to asthma management, severe asthma that is not responsive to the prescribed treatment, economic disadvantage that prevents patients from buying controller medication, insufficient resources in terms of outpatient health professionals and/or equipment and patient exposure to trigger factors (e.g.: acute respiratory infections, environmental and/or occupational irritants/allergens and tobacco consumption and/or passive exposure, among others). Usually, even patients with very frequent use of emergency care show low asthma-related hospitalization rate. However, in individuals with <5 years old and >65 years old, the number of hospitalizations has been increasing in the last two decades, especially in areas/regions with low socioeconomic development [18–20].

### Asthma co-morbidities

The most common co-morbidities related to asthma are rhinitis/rhinosinusitis, gastroesophageal reflux disease, sleep apnea, psychiatric diseases and cardiac diseases. It is estimated that more than 60% of asthmatics also have allergic rhinitis and that at least 10% have chronic sinusitis. The prevalence of other co-morbidities is lower, and it is estimated that together they do not exceed 20 to 30% in children and young adults. In elderly, however, co-morbidities are more frequent (>50%). The treatment and control of these co-morbidities, in many asthmatic patients, is essential to achieve asthma control, while keeping the focus exclusively on asthma symptoms might lead to persistent lack of disease control [1]. The concurrent treatment of asthma and its co-morbidities increases the direct costs of treatment; however, the lack of asthma control associated with deficient treatment leads to frequent emergency visits and hospital admissions, mainly in elderly people, and increases total costs of asthma management [1, 2, 5].

### Global asthma costs

Disease-related cost is usually classified into direct, indirect and intangible costs.

The studies of the economic effect of asthma have been principally of 2 kinds: those using population-based sampling frames or administrative databases to provide cost estimates for entire regions or nations, and those using clinical-based sampling frames [21]. The population-based studies have greater generalizability, whereas the clinical-based studies have greater diagnostic certainty and, frequently, data on disease severity that is particularly relevant to asthma costs.

Direct cost include asthma management (e.g. visits to emergency services; hospital admissions; medications, including all types of medications, such as over-the-counter and alternative medicines; outpatient visits, including all human resources involved, such as doctors, nurses, paramedics, psychologists…), complementary investigations or treatments (e.g. imaging, skin and blood tests, lung function tests, pulmonary rehabilitation…) and other costs (e.g. domestic or professional preventive measures, assistance in home care, transportation to medical visits…).

Indirect costs include work-related losses (e.g. temporary disability in terms of partial or total lost-days; early disability; permanent disability…) and early mortality.

Finally, intangible costs are those related with unquantifiable losses, such as the decrease in quality of life, increases in pain or suffering, limitation of physical activities and job changes. These costs, unfortunately, are not yet systematically referenced in the literature on asthma costs.

A lot of studies reported data on asthma costs, either at the individual patient, or on the society (regional or country level), and it can be an average annual per-asthmatic cost of about $USD 5,000, being much higher in severe asthmatics [21].

However, only in 2009 was published the first systematic review of the literature regarding the economic burden of asthma. The authors included 68 studies and found that hospitalization and medications were the most important drivers of direct costs; work and school loss accounted for the greatest percentage of indirect costs. Globally, asthma costs were increasing, closely correlated with comorbidities, age and asthma severity [22]. A recent estimate of total annual asthma costs in the USA, stated that they were growing fast and steadily, from $USD 12 billion in 1994 to $USD 56 billion in 2011 [23].

The total asthma costs have significant differences between countries, depending of several factors:

- 1 - Country Gross Domestic Product (GDP)
- 2 - Geographic and demographic status
- 3 - Type of health system (public and/or private)
- 3.1 - Organization of health services (health authorities, hospitals, clinics)
- 3.2 - Primary care (developed vs non-developed countries)
- 3.3 - Hospital network per region in the country
- 3.4 - Private clinics (complementary or fundamentals)
- 4 - Financial resources on Public Health
- 4.1 - Prevention and promotion of quality of life
- 4.2 - Links with organizations such as schools, pharmaceutical industry
- 4.3 - Rehabilitation of asthma patients and their work
- 5 - Relationships between governments and pharmaceutical industries regarding medications.
- 6 - Methods of data collection

According to the World Health Organization, in 2004, the world total asthma costs probably exceeded those of tuberculosis and HIV/AIDS combined [24].

The relation between direct and indirect asthma costs is variable and depends on country and type of study. Most asthma studies performed in the last 2 decades showed that direct costs are higher than indirect costs. Usually, directs costs contribute to 50 to 80% of the total costs [10, 21, 25–31].

### Direct costs

Direct cost includes asthma management and related to:

- 1.1 - Visits to emergency services
- 1.2 - Hospital admissions
- 1.3 - Medications (including all types of medications that the patient takes including OTC or use of alternative medicines
- 1.4 - Outpatient visits (includes all human resources involved, such as doctors, nurses, paramedics, psychologists, etc.)
- 1.5 - Complementary investigations or treatment (imaging, blood test, lung function tests and rehabilitation)
- 1.6 Other costs (domestic or professional preventive measures, assistance in home care, transportation to medical visits, etc.

In many countries, noncommunicable diseases, like asthma, are not yet healthcare priorities, which can lead to limited access to regular, preventive, care, both in what refers to human resources and medications. Moreover, there may be limited financial capacity to develop strategies to control and prevent this chronic disease. Therefore, in this situation, many asthmatic patients treat their asthma exclusively, or almost exclusively, on emergency rooms or during hospital admissions. One decade ago, few were the countries with a national asthma program focused on disease control, as it was the case in the USA, and in Europe in countries like Finland, France or Portugal. Beyond societal factors, such as differences in presence and type of health insurance and systems of care, there are also patient factors, like literacy, knowledge, beliefs, attitudes and language that may influence asthma costs [2].

Expenditures with asthma among USA individuals (aged 5–56 years) were compared using the 1996 to 1998 and the 2004 to 2006 Medical Expenditure Panel Surveys [32]. Direct expenditures (medications, inpatient, outpatient, and emergency services) and changes in productivity (indirect cost) were compared over this time frame. Mean annual per capita healthcare expenditures significantly increased between 1996-1998 and 2004-2006 ($3802 vs $5322), annual medication expenditures doubled from $974 to $2010 per person and outpatient visit expenditures increased from $861 to $1174, while hospitalization and emergency department visit expenditures were similar over the same period. The authors concluded that the total direct expenditures in individuals with asthma were largely driven by an increase in spending on medications [32].

Another study reporting data from Europe in a real-world evaluation of the economic cost of persistent asthma among adults per the degree of disease control (462 patients aged 30–54 years from 11 European countries, examined in clinical settings in the European Community Respiratory Health Survey II, 1999 to 2002), found that the mean total cost per patient was €1,583 and, in this study, this was largely driven by indirect costs (62.5%). The expected total cost in the population aged 30–54 years of the 11 European countries was €4.3 billion (€19.3 billion when extended to the whole European population aged from 15 to 64 years). The mean total cost per patient ranged from €509 (controlled asthma) to €2,281 (uncontrolled disease). Chronic cough or phlegm and having a high BMI significantly increased the individual total cost [14].

**Fig. 1:**
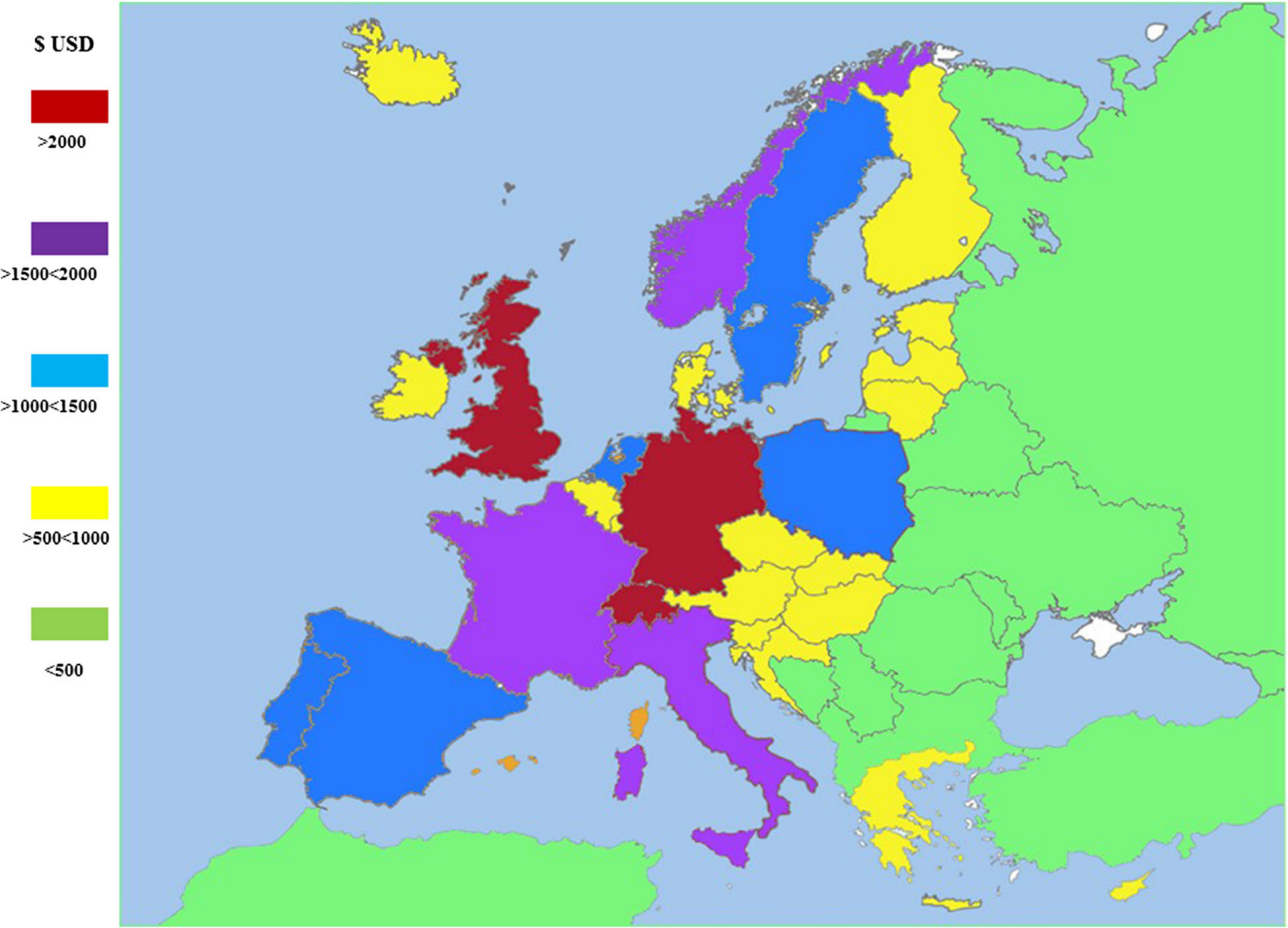
Annual cost of asthma per patient in Europe

In Fig. 1 we present an estimate of the annual asthma cost per patient in Europe using the Purchasing Power Parity (PPP) technique to make comparisons among costs in countries with different Gross National Product (GNP) and different prices on human resources, drugs and hospitalizations [33].

Within the direct costs, in some regions, like USA and several countries from Central/South America and Southeast Asia, the expenses with hospital inpatient care represent the major component [1, 22, 25]. Nevertheless, in Europe and Korea it is the outpatient medication and human resources that are responsible for most of the direct costs [10, 28, 34, 35].

In USA 2012 data from the National Hospital Ambulatory Medical Care Survey (NHAMCS), found that, annually, 20% of asthmatics, from those who need emergency service visits, have at least one hospital admission with an average of 3.6 days of hospitalization [23].

Regarding asthma-related hospitalizations, children and adolescents have a prevalence that is at least twice that of adults. In children aged 0–4 years, asthma-related hospitalizations are particularly frequent. Asthma is one of the most common chronic diseases in childhood and one of the main causes of hospitalizations [2].

The expenses related to hospital inpatient care are variable. Some studies have found higher costs in private versus public or non-profit hospitals. However, the difference is only around 20 to 30% of the cost [36–39].

In the last decade, many generic drugs to treat asthma become available, which were cheaper than brand drugs. In Europe, the prices of inhaled steroids (combined or not with long-acting bronchodilators) are variable from country to country. Most patients with persistent asthma need daily medication, have yearly differences in total medication cost among European countries.

Human resources and complementary expenses in outpatient settings, such as offices, clinics or hospital ambulatory, usually contribute from 50 to 65% of total direct costs. This percentage depends on the health system, public versus private and insurance coverage.

In Europe, for instance, there are several types of health public facility organization. We can divide them into 2 strategies: one related to “reliance on market mechanisms in service provision” and another in “mostly public provision and public insurance”. The interaction with patient payment and/or reimbursement makes a significant difference on the total direct cost supported by asthma patients and their families [40].

In UK, where there is a gate-keeping system with an ample choice of providers for users and a restrict budget constraint, asthma places a high burden on the primary health care system, with over 4 million consultations each year. An average primary care organisation in the UK including 330,000 people can expect to treat 25,000 people with asthma, with over 400 patients with asthma admitted to hospital. The total cost of asthma in UK has been estimated to be about $5 billion. This includes the cost of about $1 billion to the public health service. It is estimated that 50% of all annual healthcare costs with asthma come from the most severe asthmatic population (~20% of total individuals with asthma). About 20 million working-days are lost due to asthma each year [33].

In a cohort study of children and adolescents, followed during 20 years, it was found that patients with moderate and severe asthma had an average of 7.4 consultations per year, 3.4-fold higher than the general population needs in term of medical visits per year. Individuals with mild persistent asthma only needed an average of 3.7 medical visits per year; however, even this value is higher than the one found in the non-asthmatic population (2.1 visits). Moreover, individuals with asthma had an average of 0.7 visits per year to emergency rooms [41].

### Indirect costs

Indirect costs include:

- 1.1 Costs related to work
- 1.1.1 - Temporary disability (partial days, total days)
- 1.1.2 - Early Disability
- 1.1.3 - Permanent disability
- 1.2 - Early mortality

Asthma often generates temporary incapacity for work and a significant percentage of premature permanent disability retirements, although mortality rate is low.

The loss of productivity can be partial, with restricted work days or presentism, or total due to absenteeism from work. In most patients with asthma attacks who need home treatment, the average working day absenteeism is 5.6 days. When hospital admission occur the average number of working days lost is 13 days, with an average of 4 days of hospital stay [42–44].

Considering data from the Medical Expenditure Panel Survey in the US, over the years 2002–2007, the value of additional days lost attributable to asthma per year was approximately $301 for each worker and $93 for each student. In 2007, the total incremental cost of asthma to society was $56 billion, with productivity losses due to morbidity accounting for $3.8 billion and productivity losses due to mortality accounting for $2.1 billion [45]. Missed school and work days decreased between the 2 periods (9.2 days in 1996-1998 vs 6.4 days in 2004-2006). In the period from 2000 to 2009 it was found, in the same surveys, that although medical costs for patients with asthma increased or remained stable across all age groups over the 10-year period, outcomes did not improve; this stresses the need to reflect about the relation between asthma costs and outcomes [46].

An asthmatic child with an exacerbation of his/her symptoms, usually, loses from 3 to 5 school days and at least one of the parents/caregivers loses the same working time [2, 47, 48]. Therefore, children with asthma have more indirect costs than older asthmatics, as the parents missed work-days sum to the other expenses as an indirect cost.

**Fig. 2:**
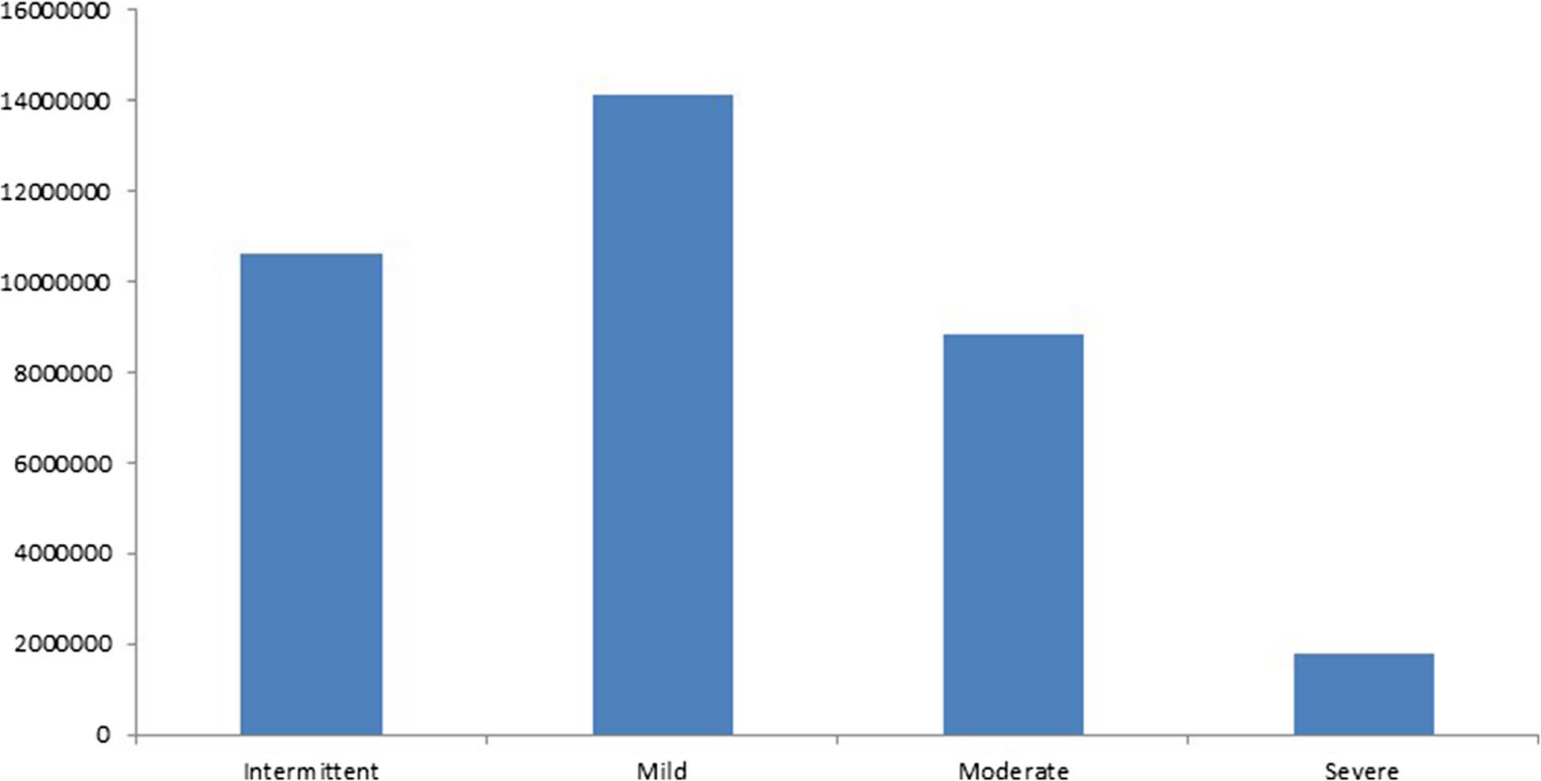
Total asthmatics in Europe by severity

**Fig. 3:**
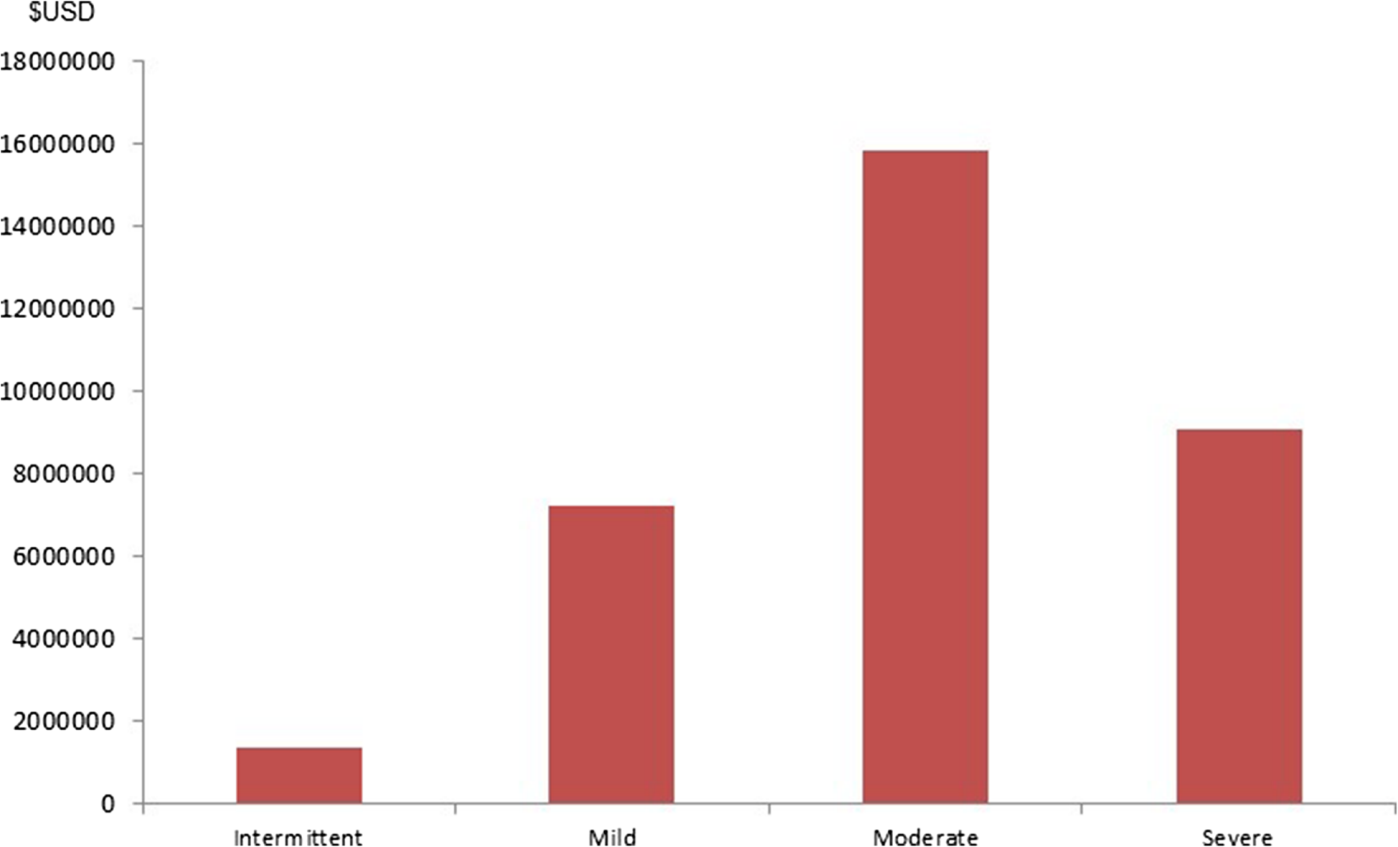
Asthma total cost by severity in Europe

Severe asthma has an estimated prevalence of up to 8% within asthmatics. Some of these patients, especially after 50 years old, can have limitations on their work and other activities due to asthma symptoms and lung function losses [49, 50]. Severe asthma, despite being less prevalent than other asthma severity classes (Fig. 2), is responsible for a large part of asthma-related costs. So, asthma cost is growing, probably more than expected, with asthma severity (Fig. 3).

Individuals with asthma can have restricted working days or, in many cases, need for temporary free days for rehabilitation of an asthma exacerbation; nevertheless, some of these patients need retirement or removal from their work due to incapacity to have a normal productivity. This contributes significantly to indirect costs and reflects on DALYs, as a measure of overall disease burden, expressed as the number of years lost due to ill-health, disability or early death. Worldwide, asthma accounts for about 1% of all DALYs lost, which simultaneously reflects high prevalence and severity [51].

Early death due to asthma is also very important. An evaluation performed by WHO refers that, in Europe, asthma is responsible for approximately 0.4% of all deaths (43,000 persons) and 1% of the global disease burden, equivalent to 1,358,000 DALYs asthma related death-rate [40].

**Fig. 4:**
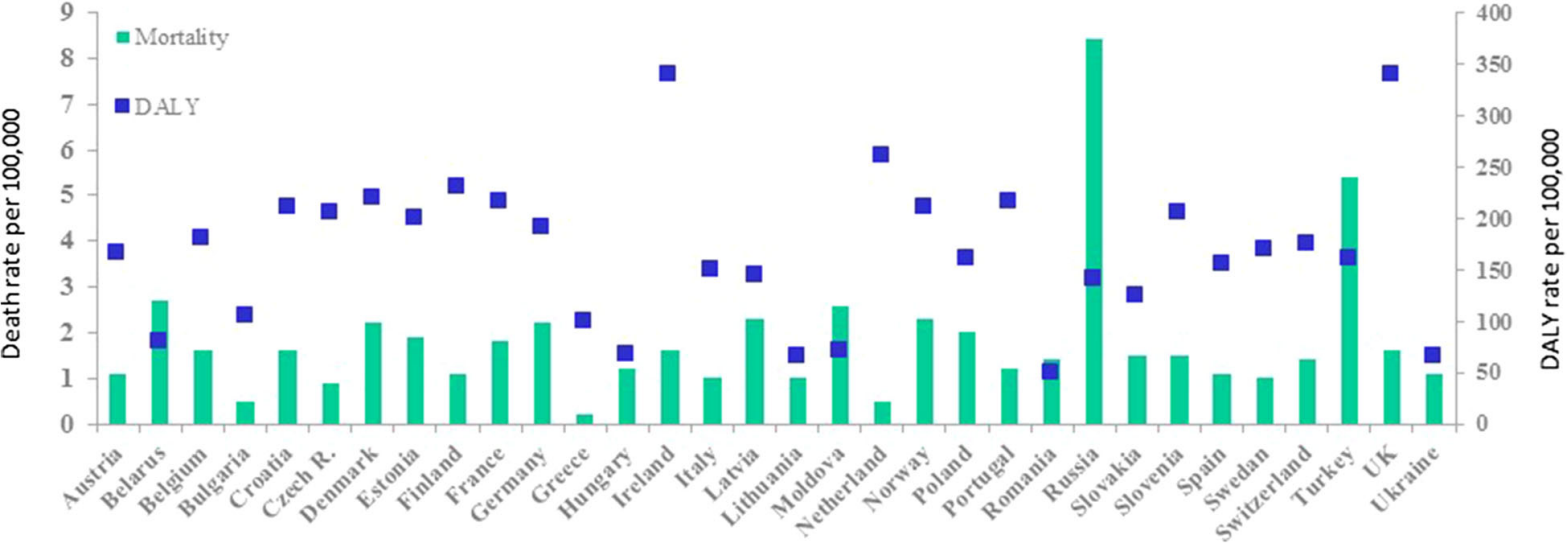
Mortality death rate and DALY rate per 100.000 inhabitants, age- standardized

Asthma-related death rate and DALYs are widely different among European countries (Fig. 4) [51].

Average life expectancy of asthmatics is lower than that of the general population. However, when interpreting these comparisons, we should consider that even within the most developed countries in the world, life expectancy at birth in men differs among European Union state members [52].

### Social impact

Socio-economic impact of a disease assessment focuses on evaluating the impacts development has on community social and economic well-being. Development impacts are generally evaluated in terms of changes in community resources, housing, employment and income, market effects, public services, and qualities of the community.

Considering this social impact related asthma costs, under economic point of view that there are significant differences among prices per country, depending of several factors:

- 1 - Country Gross Domestic Product (GDP)
- 2 - Geographic and demographic status
- 3 - Type of health system (Public versus Private)
- 3.1 - Organization of health services (health authorities, hospitals, clinics)
- 3.2 - Primary care (developed vs non-developed)
- 3.3 - Hospital network per region in the country
- 3.4 - Private clinics (complementary or fundamentals)
- 4 - Financial resources on Public health
- 4.1 - Prevention and Promotion in quality of life
- 4.2 - Links with organizations such as schools, pharmaceutical industry, etc.
- 4.3 - Rehabilitation of asthma patients and their work
- 5 - Government and pharmaceutical industry relationship regarding medication, mainly the use of generic drugs to asthma treatment.
- 6 - Methodology used to collect data

In general, in the 28 countries from the European Community, it was estimated that there are more than 30 million of asthmatics (mean asthma prevalence of 7%), that account for a total expense of more than €20 billion in the European population aged from 15 to 64 years [33].

The asthma costs are very variables from country to country, however we can estimate that a mean cost per patient per year, including all asthmatics instead of their severity (intermittent, mild, moderate and severe asthma) in Europe is $USD 1,900 [14, 33]. However, in USA, estimated mean cost per patient and per year is $USD 3,100 [8, 19, 21, 23, 32].

The assessment of asthma burden is a challenge. Even when using similar research protocols, reports on asthma prevalence and disease characterization (e.g. severity) have shown huge variations within and among countries.

Asthma prevalence and severity increased around the world, particularly in underprivileged populations. There are underserved individuals living in large urban centers of high-income countries with limited access to health care. In various large cities of low and middle-income countries, asthma prevalence is nowadays very high and most of the population cannot afford paying for the proper treatment of persistent asthma. These unfavorable scenarios lead to a situation in which dozens of millions of human beings suffering from asthma, from all age groups, cannot afford the right to breathe well [51, 53].

Despite significant progress in health outcomes over the last two to three decades, inequities continue to increase and there are gross disparities between countries at all levels of health expenditure [2].

Low-income populations, poor minorities and children living in inner cities, suffer a disproportionately higher morbidity and mortality because of asthma. In poor households, relatively small costs related with health promotion and disease control can be disastrous and assumed as catastrophic expenditures.

In children, the increasing number of hospital admissions due to asthma attacks or exacerbations, namely those related with respiratory infections, is still high, both in USA and in Europe. These contribute to increasing direct costs in hospital and medication as well as indirect costs for missing working days. In schoolchildren, there will be lost school days, which lead to limitation on study performance, with consequent psychological effects in asthmatic children.

In elderly asthmatics with several comorbidities, exacerbation of asthma symptoms is frequent, leading to increased medication need, increased outpatient hospital visits and hospital admissions, and contributing to an increase of asthma costs to the patient and society. The treatment of comorbidities can reach costs that are like asthma treatment expenses.

Management programs focused on treatment regimens which have been shown to reduce hospital admissions represent a cost-effective strategy for the management of asthma. The implementation of these programs within some developed countries, being the Finnish asthma program [54] one of the best known examples, showed a dramatic reduction in mortality and severity in parallel with costs reduction. This must also be implemented in low- and middle-income regions [1, 53].

The economic burden of asthma disparities can be decreased with access to preventive care, early treatment and the use of primary care health providers instead of emergency departments. There are some generic barriers to achieve a better health, which include poverty, poor education, lack of sanitation and poor infrastructure; and some environmental barriers that include tobacco smoking, pollution and poor nutrition. These needs to be corrected by health regional or country programs [2, 4, 11]. Moreover, the organization of health care services has inherent barriers in terms of geography, the type and training of the professionals that are needed, the type of health care to provide (public vs. private), the usual tendency for care to be “acute” rather than “routine” and the availability and use of medications.

In low income countries, asthma guidelines are strikingly hard to implement, and more research is needed to evaluate whether therapeutic trials conducted in high income countries, and resultant recommendations, are equally applicable to low income regions. Even in medium-or high-income countries are difficulties to implement national asthma plans.

Even in medium- or high-income countries, asthma burden is not seen as a health public problem and it is often poorly known.

The disease burden and health priorities differ from one country or region to another, but asthma, as other noncommunicable chronic respiratory diseases, must be in the agenda of each national authority [1, 53].

Larger studies, with comprehensive data collection, should be undertaken to make a better assessment of the magnitude of the problem and to provide data for a better-informed health policy. As an example, in the UK, an initiative aiming to provide data on previously identified gaps on asthma costs was launched; its ultimate vision is to contribute to obtain valid data that allows policy changes leading to the provision of more cost-effective care [55].

## Conclusions

Worldwide, in the last decades, asthma prevalence has been increasing. Asthma tends to be a lifelong condition with a high burden. The asthmatic patients often use emergency care, sometimes requiring hospital admission, and have a high number of school days missed and workdays lost. In addition, it can cause early permanent disability and premature death.

The economic burden of asthma is an important measure of its effect on society. Although it is recognized that asthma is a costly illness, the total cost of asthma to society has not been estimated in most countries. Asthma-related costs are high and should be systematically monitored using standardized methods; they should consider the natural history of the disease, incidence and prevalence trends, environmental impact, comorbidities, quality of life, ageing of the population, the effect of guidelines implementation and differences in national health systems and income levels.

In the last decades, several guidelines, both global (e.g. GINA) and at national/regional levels, were created trying to delineate better diagnosis and treatment strategies, emphasizing that achieving clinical control and reducing future risk should be the primary targets of asthma management; moreover, they propose approaches that promote a more adequate use of healthcare resources and should present as cost-effective strategies. However, the estimated costs of asthma can still be considered substantial, stressing the need for more comprehensive approaches that can be fully implemented in different settings.

We believe that management programs represent a cost-effective strategy for the control of asthma at a country level, and that the economic burden of asthma can be decreased with improved access to preventive care and early treatment.

## References

[1] Global Initiative for Asthma (GINA): Global strategy for asthma management and prevention. Update 2014 and Online Appendix. Available at http://www.ginasthma.org. Accessed 12 Nov 2014.

[2] Braman SS (2006). The global burden of asthma. Chest.

[3] Winer RA, Qin X, Harrington T, Moorman J, Zahran H (2012). Asthma incidence among children and adults: findings from the behavioral risk factor surveillance system asthma call-back survey—United States, 2006–2008. J Asthma.

[4] National Health Interview Survey (NHIS) 2012. Data, Statistics, and Surveillance. Available at http://www.cdc.gov/asthma/nhis/2012/data.htm. Accessed 22 Oct 2014.

[5] Masoli M, Fabian D, Holt S, Beasley R, Global initiative for asthma (GINA) Program (2004). The global burden of asthma: executive summary of the GINA dissemination committee report. Allergy.

[6] Sá-Sousa A, Jacinto T, Azevedo LF, Morais-Almeida M, Robalo-Cordeiro C, Bugalho-Almeida A, Bousquet J, Fonseca JA (2014). Operational definitions of asthma in recent epidemiological studies are inconsistent. Clin Transl Allergy.

[7] Sá-Sousa A, Morais-Almeida M, Azevedo LF, Carvalho R, Jacinto T, Todo-Bom A, Loureiro C, Bugalho-Almeida A, Bousquet J, Fonseca JA (2012). Prevalence of asthma in Portugal - The Portuguese National Asthma Survey. Clin Transl Allergy.

[8] National Health and Nutrition Examination Survey 2005–2006. http://www.cdc.gov/nchs/nhanes/nhanes2005-2006/nhanes05_06.htm.

[9] Braman SS (2003). Asthma in the elderly. Clin Geriatr Med.

[10] Lee YH, Yoon SJ, Kim EJ, Kim YA, Seo HY, Oh IH (2011). Economic burden of asthma in Korea. Allergy Asthma Proc.

[11] Asher I, Pearce N (2014). Global burden of asthma among children. Int J Tuberc Lung Dis.

[12] Bousquet J, Bousquet PJ, Godard P, Daures JP (2005). The public health implications of asthma. Bull World Health Organ.

[13] Sullivan PW, Slejko JF, Ghushchyan VH, Sucher B, Globe DR, Lin SL, Globe G (2014). The relationship between asthma, asthma control and economic outcomes in the United States. J Asthma.

[14] Accordini S, Corsico AG, Braggion M, Gerbase MW, Gislason D, Gulsvik A, Heinrich J, Janson C, Jarvis D, Jõgi R, Pin I, Schoefer Y, Bugiani M, Cazzoletti L, Cerveri I, Marcon A, de Marco R (2013). The cost of persistent asthma in Europe: an international population-based study in adults. Int Arch Allergy Immunol.

[15] The International Study of Asthma and Allergies in Childhood (ISAAC) Steering Committee (1998). Worldwide variations in the prevalence of asthma symptoms: the International Study of Asthma and Allergies in Childhood (ISAAC). Eur Respir J.

[16] World Health Organization. WHO factsheet 206: bronchial asthma. Available at: www.who.int/mediacentre/factsheets/fs206/en. Accessed 23 Oct 2014.

[17] Burney P, Jarvis D, Perez-Padilla R (2015). The global burden of chronic respiratory disease in adults. Int J Tuberc Lung Dis.

[18] Global Atlas of Asthma. Cezmi A. Akdis, Ioana Agache. Zurich, Switzerland. Edited by the European Academy of Allergy and Clinical Immunology (EAACI). 2014.

[19] Rabe KF, Adachi M, Lai CK, Soriano JB, Vermeire PA, Weiss KB, Weiss ST (2004). Worldwide severity and control asthma in children and adults. The global Asthma and Insights and Reality surveys. J Allergy Clin Immunol.

[20] Lai CK, De Guia TS, Kim YY, Kuo SH, Mukhopadhyay A, Soriano JB, Trung PL, Zhong NS, Zainudin N, Zainudin BM, Asthma Insights and Reality in Asia-Pacific Steering Committee (2003). Asthma control in the Asia-Pacific region: the Asthma Insights and Reality in Asia-Pacific Study. J Allergy Clin Immunol.

[21] Cisternas MG, Blanc PD, Yen IH, Katz PP, Earnest G, Eisner MD, Shiboski S, Yelin EH (2003). A comprehensive study of the direct and indirect costs of adult asthma. J Allergy Clin Immunol.

[22] Bahadori K, Doyle-Waters MM, Marra C, Lynd L, Alasaly K, Swiston J, FitzGerald JM (2009). Economic burden of asthma: a systematic review. BMC Pulm Med.

[23] National Hospital Ambulatory Medical Care Survey (NHAMCS), available at http://www.cdc.gov/nchs/data/ahcd/nhamcs_emergency/2010.pdf. Accessed 25 Oct 2014.

[24] http://www.who.int/mediacentre/factsheets/fs206/en/. Accessed 15 Dec 2014.

[25] Gergen PJ (2001). Understanding the economic burden of asthma. J Allergy Clin Immunol.

[26] Gendo K, Sullivan SD, Lozano P, Finkelstein JA, Fuhlbrigge A, Weiss KB (2003). Resource costs for asthma-related care among pediatric patients in managed care. Ann Allergy Asthma Immunol.

[27] Martínez-Moragón E, Serra-Batllés J, De Diego A, Palop M, Casan P, Rubio-Terrés C, Concepción Pellicer on behalf of the Asthma Cost Study Group (2009). Economic Cost of Treating the Patient With Asthma in Spain: The Asma Cost Study. Arch Bronconeumol.

[28] Kim CY, Park HW, Ko SK, Chang SI, Moon HB, Kim YY, Cho SH (2011). The financial burden of asthma: a nationwide comprehensive survey conducted in the republic of Korea. Allergy, Asthma Immunol Res.

[29] Bedouch P, Sadatsafavi M, Marra CA, FitzGerald JM, Lynd LD (2012). Trends in asthma-related direct medical costs from 2002 to 2007 in British Columbia, Canada: a population based-cohort study. PLoS ONE.

[30] Ismaila AS, Sayani AP, Marin M, Su Z (2013). Clinical, economic, and humanistic burden of asthma in Canada: a systematic review. BMC Pulm Med.

[31] Aumann I, Prenzler A, Welte T, Gillissen A (2014). Epidemiology and costs of asthma in Germany - a systematic literature review. Pneumologie.

[32] Rank MA, Liesinger JT, Ziegenfuss JY, Branda ME, Lim KG, Yawn BP, Li JT, Shah ND (2012). Asthma expenditures in the United States comparing 2004 to 2006 and 1996 to 1998. Am J Manag Care.

[33] European Lung White Book. Brussels, Belgium: European Respiratory Society and the European Lung Foundation, 2003.

[34] Borderias Clau L, Zabaleta Murguionda M, Riesco Miranda JA, Pellicer Ciscar C, Hernandez Hernandez JR, Carrillo Diaz T, Lumbreras GG (2005). Cost and management of asthma exacerbations in Spanish hospitals (COAX study in hospital services). Arch Bronconeumol.

[35] Pirina P, Carrozzi L, Dallari R, De Togni A, de Marco R (2006). Poor control increases the economic cost of asthma. A multicentre population-based study. Int Arch Allergy Immunol.

[36] Antonicelli L, Bucca C, Neri M, De Benedetto F, Sabbatani P, Bonifazi F, Eichler HG, Zhang Q, Yin DD (2004). Asthma severity and medical resource utilisation. Eur Respir J.

[37] Kiivet RA, Kaur I, Lang A, Aaviksoo A, Nirk L (2001). Costs of asthma treatment in Estonia. Eur J Public Health.

[38] Beyhun NE, Cilingiroglu N, Sekerel BE (2007). The cost of childhood asthma and its determinants in Ankara, Turkey. Turk J Pediatr.

[39] Neville RG, Hoskins G, Smith B, McCowan C (2003). The economic and human costs of asthma in Scotland. Prim Care Respir J.

[40] World Health Organization. WHO consultation on the development of a comprehensive approach for the prevention and control of chronic respiratory diseases. 2001 Geneva. Available at: www.who.int/mediacentre. Accessed 23 Oct 2014.

[41] Nunes C, Ladeira S (2004). Asthma, from childhood to adulthood, longitudinal prospective study of a cohort of asthmatics during a period of 20 year. Rev Port Imunol.

[42] Godard P, Chanez P, Siraudin L, Nicoloyannis N, Duru G (2002). Costs of asthma are correlated with severity: a 1-yr prospective study. Eur Respir J.

[43] Plaza V, Serra-Batlles J, Ferrer M, Morejon E (2000). Quality of life and economic features in elderly asthmatics. Respiration.

[44] Stock S, Redaelli M, Luengen M, Wendland G, Civello D, Lauterbach KW (2005). Asthma: prevalence and cost of illness. Eur Respir J.

[45] Barnett SB, Nurmagambetov TA (2011). Costs of asthma in the United States: 2002-2007. J Allergy Clin Immunol.

[46] Jang J, Gary Chan KC, Huang H, Sullivan SD (2013). Trends in cost and outcomes among adult and pediatric patients with asthma: 2000-2009. Ann Allergy Asthma Immunol.

[47] Meurer JR, Kuhn EM, George V, Yauck JS, Layde PM (1998). Charges for childhood asthma by hospital characteristics. Pediatrics.

[48] Huang ZJ, LaFleur BJ, Chamberlain JM, Guagliardo MF, Joseph JG (2002). Inpatient childhood asthma treatment: relationship of hospital characteristics to length of stay and cost: analyses of New York State discharge data, 1995. Arch Pediatr Adolesc Med..

[49] Serra-Batlles J, Plaza V, Morejon E, Comella A, Brugues J (1998). Costs of asthma according to the degree of severity. Eur Respir J..

[50] Hoskins G, McCowan C, Neville RG, Thomas GE, Smith B, Silverman S (2000). Risk factors and costs associated with an asthma attack. Thorax.

[51] Global Asthma Report 2014 available at http://www.globalasthmareport.org/resources/Global_Asthma_Report_2014N°978-0-473-29126-6. Accessed 12 Nov 2014.

[52] Leon DA (2011). Trends in European life expectancy: a salutary view. Int J Epidemiol.

[53] Bousquet J, Dahl R, Khaltaev N (2007). Global alliance against chronic respiratory diseases. Eur Respir J.

[54] Haahtela T, Tuomisto LE, Pietinalho A, Klaukka T, Erhola M, Kaila M, Nieminen MM, Kontula E, Laitinen LA (2006). A 10 year asthma programme in Finland: major change for the better. Thorax.

[55] Mukherjee M, Gupta R, Farr A, Heaven M, Stoddart A, Nwaru BI, Fitzsimmons D, Chamberlain G, Bandyopadhyay A, Fischbacher C, Dibben C, Shields M, Phillips C, Strachan D, Davies G, McKinstry B, Sheikh A, Burden and True Cost of Asthma in the UK Research Team (2014). Estimating the incidence, prevalence and true cost of asthma in the UK: secondary analysis of national stand-alone and linked databases in England, Northern Ireland, Scotland and Wales - a study protocol. BMJ Open.

